# Notch Signaling Limits Supporting Cell Plasticity in the Hair Cell-Damaged Early Postnatal Murine Cochlea

**DOI:** 10.1371/journal.pone.0073276

**Published:** 2013-08-30

**Authors:** Soumya Korrapati, Isabelle Roux, Elisabeth Glowatzki, Angelika Doetzlhofer

**Affiliations:** 1 Solomon H. Snyder Department of Neuroscience, Johns Hopkins University, School of Medicine, Baltimore, Maryland, United States of America; 2 Department of Otolaryngology, Head and Neck Surgery, Johns Hopkins University, School of Medicine, Baltimore, Maryland, United States of America; 3 Center for Sensory Biology, Johns Hopkins University, School of Medicine, Baltimore, Maryland, United States of America; 4 Center for Hearing and Balance, Johns Hopkins University, School of Medicine, Baltimore, Maryland, United States of America; Seattle Children's Research Institute, United States of America

## Abstract

In mammals, auditory hair cells are generated only during embryonic development and loss or damage to hair cells is permanent. However, in non-mammalian vertebrate species, such as birds, neighboring glia-like supporting cells regenerate auditory hair cells by both mitotic and non-mitotic mechanisms. Based on work in intact cochlear tissue, it is thought that Notch signaling might restrict supporting cell plasticity in the mammalian cochlea. However, it is unresolved how Notch signaling functions in the hair cell-damaged cochlea and the molecular and cellular changes induced in supporting cells in response to hair cell trauma are poorly understood. Here we show that gentamicin-induced hair cell loss in early postnatal mouse cochlear tissue induces rapid morphological changes in supporting cells, which facilitate the sealing of gaps left by dying hair cells. Moreover, we provide evidence that Notch signaling is active in the hair cell damaged cochlea and identify Hes1, Hey1, Hey2, HeyL, and Sox2 as targets and potential Notch effectors of this hair cell-independent mechanism of Notch signaling. Using Cre/loxP based labeling system we demonstrate that inhibition of Notch signaling with a γ- secretase inhibitor (GSI) results in the trans-differentiation of supporting cells into hair cell-like cells. Moreover, we show that these hair cell-like cells, generated by supporting cells have molecular, cellular, and basic electrophysiological properties similar to immature hair cells rather than supporting cells. Lastly, we show that the vast majority of these newly generated hair cell-like cells express the outer hair cell specific motor protein prestin.

## Introduction

Auditory hair cells are highly specialized mechano-sensory cells critical for our ability to perceive sound. In mammals, auditory hair cells and supporting cells are only generated once during embryonic development and loss of hair cells due to environmental stresses, ototoxicity, genetic factors, or aging is irreversible. However, non-mammalian species regenerate lost auditory hair cells. In avians, supporting cells replace lost sensory hair cells by either direct trans-differentiation [1] or by division followed by differentiation [2], [3]. It is thought that the lack of auditory hair cell regeneration in mammals is due to extrinsic factors. This is based on recent studies showing that supporting cells purified from pre-hearing neonatal mice or 2 week old hearing mice have the capacity to switch cell fate and trans-differentiate into hair cells [4]–[7].

A candidate pathway for limiting supporting cell plasticity is the Notch signaling pathway, an evolutionarily conserved cell-cell communication mechanism known to regulate sensory-neural development [8]. Canonical Notch signaling is transduced by the intracellular domain of Notch receptors (NICD). As Notch ligand binds and activates the Notch receptor, NICD is released by a series of γ-secretase dependent cleavages, which allows NICD to trans-locate to the nucleus and function as co-activator for the transcription of Notch effector genes of the Hes and Hey transcriptional repressor family [9].

During embryonic development, Notch-mediated lateral inhibition ensures that the correct number of hair cells and supporting cells are generated from a common pool of postmitotic pro-sensory progenitors. In mammals, auditory hair cell differentiation occurs in a basal to apical gradient with basal cochlear sensory progenitors differentiating first. Hair cell differentiation initiates with the up-regulation of Atoh1, a bHLH transcription factor, which is both necessary and sufficient for hair cell fate induction [10], [11]. Following Atoh1 up-regulation, the nascent hair cells start to express Notch ligands delta1 (Dll1) and jagged2 (Jag2) on their cell surface [12], [13], resulting in the activation of Notch1 receptor expressed on the cell surface of neighboring progenitor cells. Notch signaling activation in these neighboring progenitor cells prevents, through the action of the Hes/Hey Notch effectors, the up-regulation of Atoh1 and limits these cells to a supporting cell fate [14]–[17]. By E18.5, hair cell differentiation is largely completed in the murine cochlea, and the sensory epithelium is patterned into one row of inner hair cells, three rows of outer hair cells and intercalating supporting cells.

Consistent with the model of lateral inhibition, deletion of hair cell specific Notch ligands (Dll1, Jag2), or Notch1 receptor in the murine cochlea result in a dramatic overproduction of hair cells [18], [19]. As Notch signaling depends on γ-secretase activity [20], similar overproduction of hair cells can be seen, when Notch signaling is blocked using γ-secretase inhibitors (GSI) in the embryonic [21], [22] or in the early postnatal cochlea [23]–[26].

Surprisingly little is known about how Notch signaling functions in the absence of hair cells. It has been proposed that the supporting cell specific Notch ligand jagged1 (Jag1) might be involved in the maintenance of supporting cells [27]. Prior to auditory hair cell differentiation, Notch ligand Jag1 functions in pro-sensory cell specification [19], [28]. At later stages, Jag1 expression is restricted to differentiating supporting cells and is believed to function in supporting cell maintenance [29]. To better understand the role of Notch signaling in the hair cell-damaged cochlea, we developed a hair cell ablation and hair cell regeneration assay using a well characterized early postnatal cochlear explant culture system. Our experiments demonstrate that Notch signaling is active in the hair cell-depleted cochlea, and that Hes1, Hey1, Hey2, HeyL, and Sox2 are targets and likely effectors of this hair cell-independent mechanism of Notch signaling. Using Cre/LoxP based labeling, we demonstrate that prolonged inhibition of Notch signaling results in the trans-differentiation of supporting cells into hair cell-like cells. Moreover, we provide evidence that these postnatally formed hair cell-like cells exhibit molecular and cellular characteristics of immature hair cells and exhibit basic electrophysiological properties similar to immature hair cells rather than supporting cells. Lastly, we show that the vast majority of these newly generated hair cell-like cells express the outer hair cell specific motor protein prestin.

## Materials and Methods

### Ethics Statement

All experiments and procedures were approved by the Johns Hopkins University Institutional Animal Care and Use Committees protocol and all experiments and procedures adhere to NIH-approved standards.

### Mouse Breeding and genotyping

Atoh1/nGFP transgenic line was obtained from Jane Johnson (UT Southwestern Medical Center, Dallas) [30]. Lfng/GFP BAC transgenic mouse line was generated by GENSAT project [31] and was obtained from Nathaniel Heintz (Rockefeller University, New York). Prox1-CreER transgenic mouse line [32] was obtained from Guillermo Oliver (St. Jude Children's Research Hospital, Memphis) and mTmG Cre reporter line [33] was purchased from Jackson Laboratories, stock# 007576. Mouse strains were maintained on a CD1 background. The following primers were used for PCR based genotyping: Lfng/GFP and Atoh1/nGFP: GFP-F CGA AGG CTA CGT CCA GGA GCG CAC CAT; GFP-R GCA CGG GGC CGT CGC CGA TGG GGG TGT TCT GC. Prox1-CreER: Cre-F AAC ATG CTT CAT CGT CGG TCC GGG CTG C, Cre-R GAC GGA AAT CCA TCG CTC GAC CAG TTT A. Cre reporter mTmG: mTmG wt-F CTC TGC TGC CTC CTG GCT TCT, mTmG wt-R CGA GGC GGA TCA CAA GCA ATA, mTmG-mt TCA ATG GGC GGG GGT CGT T.

### Organotypic cultures

The culture procedures were modified from published reports [24]. In brief, cochleae from postnatal day 2 (P2) mice were harvested in Hank's Balanced Salt Solution (HBSS, Life Technologies). To obtain a flat cochlear surface preparation, the spiral ganglia, Reissner's membrane, and the most basal cochlear segment (0.5 mm) were removed. For q-PCR experiments, both the most cochlear base and the cochlear apex were removed. The remaining cochlear tissue containing the auditory sensory epithelium was placed onto filter membranes (Spi Supplies) and cultured with DMEM-F12 (Life Technologies), 1x N2 supplement (Life Technologies), 5 ng/ml EGF (Sigma) and 100 U/ml Penicillin (Sigma). All cultures were maintained in a 5% CO_2_/ 20% O_2_-humidified incubator.

### Hair cell ablation

Gentamicin sulfate solution (G1272, Sigma) was added to cochlear cultures at plating and was washed out after 14 hours. If not stated otherwise, gentamicin was used at a final concentration of 100 μg/ ml (∼0.22 mM).

### Notch inhibition

DAPT (γ-secretase inhibitor IX, Calbiochem-EMD Biosciences) was stored as a 25 mM stock in DMSO at −80°C and was used at a final concentration of 10 μM. DAPT or vehicle control (0.04%) was added after 14 hours of plating.

### EdU incorporation assay

EdU (5-ethynyl-2′-deoxyuridine, Life Technologies) was reconstituted in DMSO (100 mM stock solution) and used at a final concentration of 3 μM. Click-iT Alexa Fluor 488 Kit (Life Technologies) was used to detect incorporated EdU. The assay was performed according to manufacturer's specifications.

### RNA extraction and real-time PCR

For RNA extraction, cochlear epithelia were isolated from cultured cochlear explants using dispase (1 mg/ml; Invitrogen) and collagenase (1 mg/ml; Worthington) as previously described [24]. For each experiment 3–4 cochlear epithelia were pooled per condition and RNeasy Micro kit (QIAGEN) was used to isolate total RNA and mRNA was reversed transcribed into cDNA using iScript kit (Bio-Rad). Q-PCR was performed using SYBR Green kit (Life Technologies) and gene specific primer sets on a StepOne Plus PCR Detection System (Applied Biosystems® Life Technologies). Each PCR reaction was carried out in triplicate. Relative gene expression was analyzed by using the ΔΔCT method [34]. The comparative Ct study feature of StepOne plus software (Applied Biosystems® Life Technologies) was used to aggregate biological replicate data. cDNA from one of the control samples was used as a calibrator, and the ribosomal gene Rpl19 was used as endogenous reference gene. Values are presented as mean ± standard error (SEM), n = biological replicates analyzed. Two-tailed *Student's* t-tests were used to determine confidence interval. *P*-values equal or less than 0.05 were considered significant. The following primers were used for q-PCR:

Atoh1-F ATGCACGGGCTGAACCA; Atoh1-R TCGTTGTTGAAGGACGGGATA

Fgf8-F ATCAACGCCATGGCAGAA G; Fgf8-R AGTATCGGTCTCCACAATGAGCTT

Hey1-F CACTGCAGGAGGGAAAGGTTAT; Hey1-R CCCCAAACTCCGATAGTCCAT

Hey2-F AAGCGCCCTTGTGAGGAAA; Hey2-R TCGCTCCCCACGTCGAT

HeyL-F GCGCAGAGGGATCATAGAGAA; HeyL-R TCGCAATTCAGAAAGGCTACTG

Hes1-F GCTTCAGCGAGTGCATGAAC; Hes1-R CGGTGTTAACGCCCTCACA

Hes5-F GGCGGTGGAGATGCTCAGT; Hes5-R GCTGCTCTATGCTGCTGTTGA

Jag1-F TGTGCAAACATCACTTTCACCTTT; Jag1-R GCA AAT GTG TTC GGT GGT AAG AC

Nhlh1-F TGCCCCCGGACAAGAAG; Nhlh1-R TGGTTCAGGTAGGAGATATAGCAGATG

Ocm-F ACCAGAGTGGATACCTGGATGAA; Ocm-R CGTCGCTCTGGAACCTCTGT

Otof-F CCG CTC TCA CCA GCA ATG T Otof-RGCA CTG GGC TCC ATC TTA ATA TCT

Pou4f3-F GCACCATCTGCAGGTTCGA; Pou4f3-R CCGGCTTGAGAGCGATCA T

Prox1-F CGTTACGGGAGTTTTTCAATGC; Prox1-R CCTTGTAAATGGCCTTCTTCCA

Rpl19-FGGTCTGGTTGGATCCCAATG; Rpl19-R CCCGGGAATGGACAGTCA

S100a1-F TGGATGTCCAGAAGGATGCA; S100a1-R CCGTTTTCATCCAGTTCCTTCA

Sox2-F CTGTTTTTTCATCCCAATTGCA; Sox2-R CGGAGATCTGGCGGAGAATA

Strc-F TCTGGCAAACCGAAGACTCTATC; Strc-R CACTTGCATTTTCTTCCAGAGAAA

Slc26a5-F ACGATCTCCATGGCCAAAAC; Slc26a5-R GAGCTCCTGATTGCCATCAAC

Tmc1-F GGCCAAGAAATGGGCAAAAT; Tmc1-R CGCAAGCCGCTTTGAAGT

Tmc2-F CCTCATTTACTTTGTGGTGAAACG; Tmc2-R TCATACCAGCTGACATTTTGCAT.

### Cell Counts

Hair cells were identified by myosin VI and Atoh1/nGFP co-expression. Composite images of cochlear explants were assembled and analyzed in PhotoShop CS3 (Adobe). The cochlear apex (1 mm) was excluded from our analysis. Number of hair cells was counted for mid-apex (1.5 mm) only or separately for mid-apex (1.5 mm), mid-base (1.5 mm) and base (1 mm). ImageJ software (NIH) was used to measure the length of analyzed cochlear segments and hair cell density (cells per 100 micron) was then calculated for each segment. Values are presented as mean ± standard error (SEM) (n = 3, cochlear explants per condition were analyzed from two independent experiments). Two-tailed unpaired *Student's* t-tests were used to determine confidence interval. *P-*values equal or less than 0.05 were considered significant.

### Immuno-histochemistry

Antibodies used in this study were anti-myosin VI (1∶1000, Proteus), anti-parvalbumin, clone PARV-19 (1∶3000, Sigma), anti-prestin (1∶500, Santa Cruz), anti-Prox1 (1∶1000, Chemicon), anti-Sox2 (1∶1000, Santa Cruz). Cell nuclei were fluorescently labeled with Hoechst-33258 (Sigma). Actin rich stereocilia were labeled with fluorescently labeled phalloidin 488 (Life Technologies). Alexa Fluor (488, 546 and 633) labeled secondary antibodies (1∶1000, Life Technologies) were used.

### Electrophysiological recordings

Cochlear preparations, either acutely isolated or after 6 to 7 days in culture, were placed into a chamber under an upright microscope (Axioskope, Zeiss, Oberkochen, Germany) and superfused at 2–3 ml/minute (chamber volume ∼2 ml), with an external solution containing (in mM): 5.8 KCl, 144 NaCl, 1.3 CaCl_2_, 0.9 MgCl_2_, 0.7 NaH_2_PO_4_, 5.6 D-glucose and 10 HEPES (300 mOsm, pH 7.4 (NaOH)). nGFP positive hair cells, nGFP positive hair cell-like cells (bearing stereocilia-like hair bundles and localized in clusters) and supporting cells (nGFP negative) were identified by fluorescence microscopy and visualized on a monitor via a 40x water immersion objective, 4x magnification, differential interference contrast optics using a green filter, and camera with contrast enhancement (NC70 Newvicon camera (Dage MTI)). The internal solution contained (in mM): 135 KCl, 3.5 MgCl_2_, 0.1 CaCl_2_, 5 EGTA, 5 HEPES, 2.5 Na_2_ATP (290 mOsm, pH 7.2 (KOH)). Recording pipettes were fabricated from 1 mm borosilicate glass (WPI, Sarasota, FL) and coated with Sylgard® (Dow Corning, Midland, MI). Pipettes were pulled with a multi-step horizontal puller (Sutter, San Rafael, CA), pipette resistances were ∼ 2–6 MΩ. Recordings were performed at room temperature (22–25° C). Currents were recorded with an Axopatch 200B amplifier (Molecular Devices, Union City, CA), low-pass filtered at 10 kHz and digitized at 20–50 kHz with a Digidata 1440A board. Data were stored with PClamp10.3 software (Molecular Devices). Voltages were corrected off-line for liquid junction potentials (∼−4 mV). Membrane capacitance (Cm), series resistance (Rs) and membrane resistance (Rm), were calculated from the average current response to 10 mV steps, from −84 to −94 mV, or from −70 to −80 mV for the latter. Rs values were 11.5±1.1 MΩ (n = 24) and were not compensated for. Only recordings with holding currents <175 pA at a holding potential of −70 mV were included. Current amplitudes were adjusted off line using leak subtraction. Data were analyzed off-line using Clampfit 10.3 (Molecular Devices) and Origin (OriginLab Corporation) software. Values are presented as mean ± standard error of mean (SEM) with n representing the number of recordings. Statistical analyses were performed by One-way ANOVA followed by Bonferroni's post hoc test for multiple comparisons (significance, α<0.05) or by two tailed two sample t-test equal variance not assumed. *P*-value less than 0.05 were considered significant.

## Results

### Gentamicin allows for fast and efficient hair cell ablation in the early postnatal cochlea

**Figure 1:**
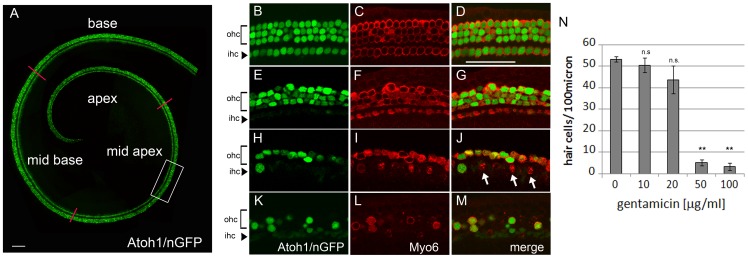
Gentamicin exposure results in rapid inner and outer hair cell loss. **A:** Acutely isolated cochlear explant (stage P2). Atoh1/nGFP expression (green) labels inner and outer hair cell nuclei. Red lines subdivide auditory sensory epithelium into apex, mid apex, mid base and base. Scale bar 100 μm. **B–M:** Hair cell phenotype 24 hours after exposure to various concentrations of gentamicin. Confocal images are taken from the central part of mid apical segment (A, white box). Brackets mark outer hair cell domain (ohc), arrowheads point to inner hair cell domain (ihc). Myosin VI antibody staining (Myo6, red) labels cytoplasmic domain of hair cells. Atoh1/nGFP transgene (green) labels hair cell nuclei. Scale bar 50 μm. **B–D**: control (0 μg/ml gentamicin). **E–G:** 10 μg/ml gentamicin. **H–J:** 50 μg/ml gentamicin. White arrow marks Myo6 positive hair cell debris. **K–M**: 100 μg/ml gentamicin. **N:** Quantification of B-M. Hair cell numbers were analyzed in the mid apical segment. Data expressed as mean ±SEM (n = 3 cochlear explants from 2 independent experiments; ** p≤0.0001, *Student's* t-test; *p*>0.05 was considered not significant (n.s.)).

Hair cells are highly sensitive to amino-glycosides, and the amino-glycoside gentamicin has been used in ototoxicity research in various animal models [35]. To determine the optimal concentration of gentamicin, which yields both fast and selective hair cell ablation in the early postnatal cochlea, postnatal day 2 (P2) cochlear explants were exposed for 14 hours to different concentrations of gentamicin and maintained for an additional 10 hours after gentamicin removal. The hair cell specific Atoh1/nGFP reporter line was used to monitor the dynamics of hair cell loss [30]. Hair cells localized to the cochlear apex are resistant to gentamicin in the early postnatal cochlear tissue and were excluded from our analysis [36] (Fig. 1 A; Fig. S1 A). Low concentrations of gentamicin (10–20 μg/ml) resulted in altered hair cell morphology, but yielded only modest hair cell loss within 24 hours (Fig. 1 E–G, N), whereas higher gentamicin concentrations (50–100 μg/ml) resulted in rapid loss of both inner and outer hair cells throughout the cochlear sensory epithelium (Fig. 1 H–M, N). Exposure to gentamicin at a concentration of 100 μg/ml for 14 hours killed 90% of hair cells in the cochlear mid apex within 24 hours (Fig. 1 K–M, N; Fig. S1 A). This hair cell ablation paradigm was used in all subsequent experiments.

### Supporting cell survival is only modestly affected in the hair cell depleted cochlea

**Figure 2:**
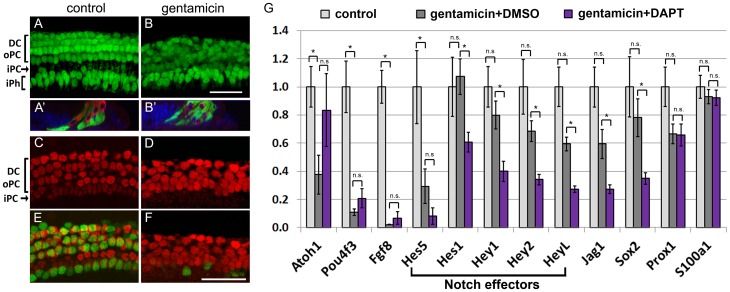
Supporting cell phenotype in the hair cell damaged cochlea. **A–B**': P2 Lfng/GFP transgenic control (A, A') and gentamicin treated (B, B') cochlear cultures after 1 DIV. Deiters cell (DC), outer pillar cell (oPC) and inner phalangeal cell nuclei (iPh) are visualized by native Lfng/GFP expression (green); hair cells are immuno-stained with myosin VI antibody (Myo6, red). **A, B:** Confocal images of supporting cell layer. **A**' **B**'**:** orthogonal section of z-stack projections of supporting cell and hair cell layer. 24 hours after initial gentamicin insult, Lfng/GFP positive supporting cell layer is disorganized (B) and myosin VI positive hair cell debris (red) is engulfed by Lfng/GFP positive supporting cells (green) (B'). DAPI (blue) labels cell nuclei. Scale bar 50 μm. **C–F:** Confocal images of P2 Atoh1/nGFP transgenic control (C, E) and gentamicin treated (D, F) cultures after 2 DIV. Prox1 immuno-staining (red) marks Deiters cells (DC), outer pillar cells (oPC) and inner pillar cell (iPC) nuclei and Atoh1/nGFP expression (green) marks hair cell nuclei. Distinct oval nuclear morphology of inner pillar cells seen in control cultures (C) is largely lost in gentamicin treated cultures (D). Scale bar: 50 μm. **G:** Relative mRNA levels of Notch effector genes (bracket) and Notch target genes (Jag1, Sox2) were analyzed in undamaged (control, white bar), hair cell damaged (gentamicin +DMSO, grey bar) and hair cell damaged and Notch inhibited (gentamicin +DAPT, purple bar) cochlear epithelia after 38 hours in culture using q-PCR. Gentamicin was added at plating and washed out after 14 hours of culture. DAPT or vehicle control DMSO was added following gentamicin treatment for 24 hours. Hair cell specific genes (Atoh1, Pou4f3, and Fgf8) and supporting cell specific genes (Prox1, S100a1) were analyzed as experimental controls. Data expressed as mean ±SEM (n = 5, independent experiments analyzed * *p*≤0.05, *Student's* t-test, *p*>0.05 was considered not significant (n.s)).

Several morphologically and functionally distinct types of supporting cells (inner phalangeal cells, Deiters cells, inner and outer pillar cells) exist in the murine cochlea [37]. Recent studies examining the effects of aminoglycosides *in vivo* reported varying degrees of loss of these supporting cell subtypes in the hair cell-damaged mammalian cochlea [27], [38], [39]. To monitor morphological changes and supporting cell viability, a bacterial artificial chromosome (BAC) transgenic reporter mouse line Lfng/GFP, obtained from the Gene Expression Nervous System Atlas (GENSAT) project was used [31]. Similar to endogenous Lfng, Lfng/GFP is expressed in inner phalangeal cells, which surround inner hair cells, and in Deiters and outer pillar cells, which surround outer hair cells, but is not expressed in inner pillar cells [40] (Fig. 2 A). 24 hours after the initial gentamicin insult, supporting cells had filled in the voids left by hair cells (Fig. 2 B'). Moreover, hair cell debris deep in the supporting cell layer, seemingly engulfed by supporting cells was observed (Fig. 2B' ). Acute survival of supporting cells was only modestly impacted by gentamicin treatment, with 79%± 5% (n = 3 cochlear explants) of Lfng/GFP-positive supporting cells surviving the first 24 hours (Fig. 2 A, B). However, we noted that the supporting cell layer was disorganized in gentamicin treated cultures and that Lfng/GFP positive inner phalangeal cells and Lfng/GFP positive outer pillar cells, normally separated by Lfng/GFP negative inner pillar cells, were in close proximity (Fig. 2 A, B). To analyze the inner pillar cell phenotype in hair cell-damaged cochlea, gentamicin treated cochlear explants and control explants were immuno-stained with Prox1 antibody. In the early postnatal cochlea, the transcription factor Prox1 is highly expressed in inner pillar cells as well as in outer pillar cells and Deiters cells and Prox1 positive inner pillar cells can be readily identified by their distinct oval nuclear shape [41] (Fig. 2 C, E). In the hair cell-ablated epithelium, Prox1 positive nuclei lacked the stereotypical arrangement seen in control cultures, nuclei appeared enlarged, and oval shaped nuclei typical for inner pillar cells were missing (Fig. 2 C–F). As inner pillar cells depend on Fgf8-Fgfr3 signaling it is likely that the gentamicin induced loss of Fgf8 expression (Fig. 2 G), and the subsequent loss of Fgf8-Fgfr3 signaling in hair cell ablated cultures caused inner pillar cells to de-differentiate [42]–[44]. Alternatively, it is also possible that the used gentamicin concentration was toxic to inner pillar cells resulting in inner pillar cell death in gentamicin treated cochlear explants.

### Notch effector genes continue to be expressed in the hair cell depleted cochlea

Lateral inhibition, mediated by hair cell specific Notch ligands (Dll1, Jag2) is believed to be the main mechanism of Notch signaling activation in the differentiating cochlea [18], [19]. Given the importance of hair cell mediated Notch signaling, surprisingly little is known about how acute hair cell loss might affect Notch signaling activation in supporting cells. To test if Notch signaling pathway is active in the hair cell-depleted early postnatal cochlea, Notch effector (Hes1, Hes5, Hey1, Hey2, HeyL) [14], [24], [45] and Notch target (Sox2, Jag1) [46]–[48] genes were analyzed in undamaged cochlear cultures, and in hair cell-damaged cultures, which following 14 hours of gentamicin exposure, received Notch inhibitor DAPT or DMSO for 24 hours (Fig. S1 C, D).

Consistent with the almost complete loss of both inner and outer hair cells, expression levels of hair cell specific transcription factor Pou4f3 [49] and the inner hair cell specific Fgf ligand Fgf8 [42] were reduced by 90% in gentamicin treated cultures compared to control cultures (Fig. 2 G, gentamicin +DMSO vs. control). Interestingly, expression of hair cell specific transcription factor Atoh1 was less severely reduced (∼70%) after gentamicin treatment (Fig. 2 G, gentamicin +DMSO vs. control). Atoh1 expression in supporting cells and non-sensory epithelial cells is inhibited by Notch effectors of the Hes and Hey family [16], [24]. The slightly higher than expected expression of Atoh1 in the hair cell-damaged cochlea might have been caused by a rise of Atoh1 in the remaining few hair cells as part of a survival mechanism. Alternatively, the slightly higher than expected Atoh1 levels in hair cell damaged cochlea tissue might be due to the loss of hair cell-mediated Notch signaling in supporting cells. Consistent with the loss of hair cell-mediated Notch signaling, expression of Notch effector gene Hes5, which in the developing cochlea relies on hair cell specific Notch ligand Jag2 for its expression [13], was reduced by ∼70% after 38 hours *in vitro* (Fig. 2 G gentamicin + DMSO vs. control).

In contrast to Hes5, other supporting cell specific genes (Prox1, S100a1), including the remaining Notch effector and Notch target genes (Hes1, Hey1, Hey2, HeyL, Sox2, and Jag1) were only modestly reduced in hair cell-damaged cochlear epithelia (Fig. 2 G, gentamicin + DMSO vs. control). However, hair cell-damaged cochlear cultures, which received Notch inhibitor DAPT for 24 hours expressed Hes1, Hey1, Hey2, HeyL, Sox2 and Jag1 at significantly lower levels than hair cell-damaged cochlear cultures treated with DMSO (Fig. 2 G, gentamicin +DMSO vs. gentamicin +DAPT), indicating that in the hair cell-damaged cochlea the expression of Hes1, Hey1, Hey2, HeyL, Jag1 and Sox2 is maintained by a Notch dependent mechanism. In summary, these findings demonstrate that Notch signaling is still active in the hair cell damaged cochlea and identify Hes1, Hey1, Hey2, HeyL, and Sox2 as targets and potential Notch effectors of this hair cell-independent mechanism of Notch signaling.

### Notch inhibition in the hair cell damaged cochlea allows for the production of new hair cell-like cells

**Figure 3:**
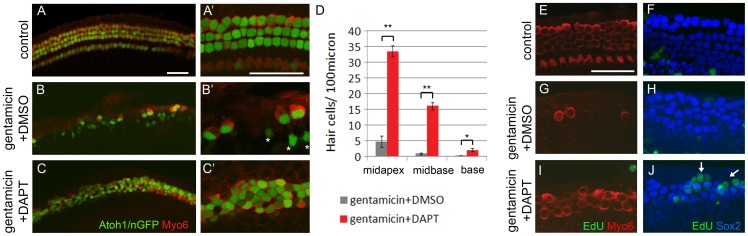
Inhibition of Notch signaling allows for hair cell regeneration in gentamicin damaged cochlea. **A–C:** Hair cell phenotype in control (A, A'), gentamicin +DMSO (B, B') and gentamicin +DAPT (C, C') treated cultures after a total of 3 DIV. Atoh1/nGFP expression (green) labels hair cell nuclei and myosin VI immuno-staining marks hair cells (Myo6, red). All images shown are from the cochlear mid apical region. **A**'**–C**': higher magnification of A–C. Asterisk in B' mark faint nGFP expression in inner phalangeal cells. Scale bar 50 μm. **D:** Quantification of hair cell density in mid apex, mid base and base of gentamicin + DMSO (grey) and gentamicin +DAPT (red) treated cochlea cultures after 3 DIV. Data expressed as mean ±SEM (n = 3, cochlea explants from 2 independent experiments; ** *p-*value <0.005; * *p-*value ≤0.05). **E–J:** EdU (green) incorporation in control (E, F), gentamicin +DMSO (G, H) and gentamicin +DAPT (I, J) treated cultures. All images shown are from the cochlear mid apical region. **E, G, I:** Confocal images of hair cell layer. Myosin VI immuno-staining marks hair cells (Myo6, red). **F, H, J**: Projections of confocal z-stacks, imaging supporting cell layer. Sox2 immuno-staining (blue) marks supporting cells. No EdU positive hair cell (Myo6, red) or EdU positive supporting cell nuclei (Sox2, green) are observed in control (E, F), gentamicin (G, H), or gentamicin +DAPT (I, J) treated cochlea cultures. White arrows in J mark EdU positive cells at the lateral edge of sensory epithelium. Scale bar 50 μm.

**Figure 4:**
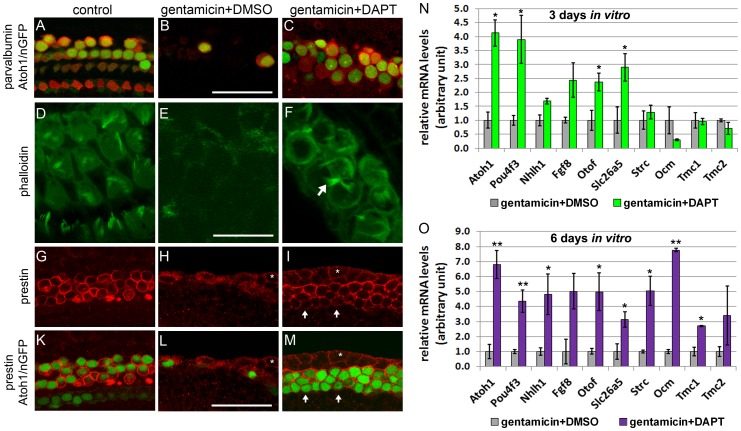
Newly generated hair cell-like cells mature *in vitro*. Hair cell phenotype of untreated (control A, D, G, K) gentamicin +DMSO (B, E, H, L) and gentamicin +DAPT (C, F, I, M) treated cochlear explants. **A–C:** Parvalbumin immuno-staining (red) and native Atoh1/nGFP (green) expression mark hair cells. After 3 DIV, Atoh1/nGFP positive cells (green nuclei) in gentamicin + DAPT treated (C) cultures express hair cell marker parvalbumin (red). Scale bar 50 μm. **D–F:** Fluorescently labeled phalloidin (green) visualizes actin. After a total of 3 DIV, gentamicin + DAPT (F) treated cultures have actin-rich (phalloidin, green) membrane protrusions (white arrows) reminiscent of hair cell stereocilia. Scale bar 25 μm. **G–M**: Prestin immuno-staining (red) selectively marks outer hair cells. After 6 DIV, the majority of Atoh1/nGFP positive cells co-express prestin in gentamicin + DAPT treated cultures (I, M). Note white asterisk labels Atoh1/nGFP negative epithelial cells at the lateral edge of cochlear epithelium, which express prestin at low levels (H, I, L, M). Scale bar 50 μm. **N–O**: Q-PCR based quantification of mRNA expression levels of hair cell specific genes in hair cell damaged (gentamicin +DMSO) and hair cell damaged and Notch inhibited (gentamicin +DAPT) cochlear epithelia after 3 DIV (N) and 6 DIV (O). Data expressed as mean ± SEM, (n = 3 independent experiments, *p≤0.05, *Student's* t-test).

During cochlear development Hes1, Hes5 and Hey factors function as “hair cell-fate repressors”. Combined loss of Hes1, Hes5 and Hey factors result in hair cell overproduction and severe patterning defects [14], [15], [17]. We reasoned that sustained inhibition of Notch signaling would reduce Hes and Hey expression to a level low enough to allow for new generation (regeneration) of hair cells. To test our hypothesis we cultured untreated (control) or gentamicin treated Atoh1/nGFP transgenic cochlear tissue in the presence of GSI (DAPT) or vehicle control (DMSO) for a total of 3 days (3 DIV). After 3 DIV, less than 10% of inner and outer hair cells remained in gentamicin treated cultures (Fig. 3 B–B'), whereas control cultures maintained the normal compliment of one row of inner and three rows of outer hair cells (Fig. 3 A–A'). In the first 38 hours of culture, hair cell-damaged cochlear explants, treated with GSI DAPT, were indistinguishable from hair cell-damaged cochlear explants treated with DMSO, retaining only few scattered hair cells that survived the gentamicin insult (Fig. S1). However, after 3 DIV, inhibition of Notch signaling resulted in the new generation of densely packed Atoh1/nGFP positive cells within the sensory region (Fig. 3 C–D). A robust increase in the number of Atoh1/nGFP positive hair cell-like cells was observed throughout in the mid-turn of the cochlear duct (mid-apex, mid-base) and a modest increase in the cochlear base (Fig. 3 D). The newly formed Atoh1/nGFP positive cells in gentamicin +DAPT treated cultures co-expressed the hair cell markers myosin VI [50] (Fig. 3 C, C'), and parvalbumin [51] confirming their hair cell identity (Fig. 4 A, C). In addition, more than 80% of these hair cell-like cells displayed actin-rich apical membrane protrusions reminiscent of very immature cochlear hair cell bundles, but lacked the “V” shaped hair cell bundle organization of early postnatal cochlear hair cells [52] (Fig. 4 D, F).

To determine if the newly generated hair cell-like cells originated from dividing cells, EdU (5-ethynyl-2′-deoxyuridine) labeling experiments were performed. EdU is a thymidine analog, which in dividing cells is incorporated into newly synthesized DNA of dividing cells. After 3 DIV, no EdU labeled myosin VI positive hair cells were observed in control cultures and hair cell-ablated cultures treated with GSI (DAPT) or vehicle control (DMSO) (Fig. 3 E, G, I). The lack of EdU labeling indicates that the newly generated hair cell-like cells originated from post-mitotic cells (Fig. 3 I). We frequently observed EdU positive cells at the lateral edge of the Sox2 positive supporting cell domain in the Notch inhibited hair cell-ablated tissue (Fig. 3 J, white arrows). However, we did not observe Sox2 EdU double positive supporting cells in untreated, or gentamicin treated cultures with and without GSI (DAPT) (Fig. 3 F, H, J). This finding suggests that similar to the intact cochlea, inhibition of Notch signaling in the hair cell-damaged cochlea has no immediate effect on the proliferative state of supporting cells and supporting cells remain post-mitotic [24].

### Newly formed hair cell-like cells mature *in vitro* and show subtype specific specialization

To determine to what extent these newly formed hair cell-like cells differentiate and mature *in vitro*, “early” and “late” hair cell specific genes were analyzed. The group of hair cell specific genes analyzed also included genes selectively expressed in either outer hair cells or inner hair cells. Outer and inner hair cells are structurally and functionally distinct types of mechano-sensory cells with unique gene expression profiles [53]. Little is known about the underlying mechanisms that determine inner or outer hair cell fate during development and it is unknown if subtype specific specialization can occur when hair cells are produced at postnatal stages. To determine if the newly generated hair cell-like cells differentiate and undergo subtype specific specialization *in vitro*, hair cell-ablated cultures were maintained for either 3 or 6 DIV with GSI (gentamicin +DAPT) or vehicle control (gentamicin +DMSO) and mRNA expression of genes expressed “early” or “late” during hair cell development were analyzed using quantitative PCR (q-PCR). All “early” hair cell specific genes examined (Atoh1, Pou4f3, Nhlh1, otoferlin (Otof) and Fgf8) are induced *in vivo* within 3 days of hair cell-fate induction [42], [54]–[56]. “Late” hair cell specific genes examined were trans-membrane channel 1 (Tmc1) and trans-membrane channel 2 (Tmc2) [57], the outer hair cell specific genes stereocilin (Strc) [58], prestin (Slc26a5) [59] and oncomodulin (Ocm) [60], which in the murine cochlea are induced within 5-8 days of hair cell-fate induction. After 3 DIV, newly generated hair cell-like cells (gentamicin and DAPT) expressed early hair cell specific genes (Atoh1, Pou4f3, Nhlh1, Fgf8, Otof) but showed little (Slc26a5) or no induction of “late” hair cell specific genes (Tmc1, Tmc2, Strc, Ocm) (Fig. 4 N). However, after 6 DIV both “early” and “late” hair cell specific genes were up-regulated in Notch inhibited cultures (gentamicin +DAPT) compared to control cultures (gentamicin +DMSO) (Fig. 4 O), suggesting that our culture system allows newly formed hair cell-like cells to differentiate and mature *in vitro*.

We observed up-regulation of both inner (Fgf8) and outer (Slc26a5, Ocm, Strc) hair cell specific genes, suggesting that the newly formed hair cell-like cells might initiate a subtype specific hair cell program (Fig. 4 O). To determine what percentage of newly formed hair cell-like cells expressed the outer hair cell specific motor protein prestin, immuno-staining with a prestin specific antibody was performed. Consistent with prestin marking only outer hair cells, approximately 75% of Atoh1/GFP positive hair cells co-expressed prestin in our control cultures (Fig. 4 G, K). However, more than 90% of Atoh1/nGFP positive hair cell-like cells co-expressed prestin in gentamicin +DAPT treated cultures (n = 3, cochlea cultures), suggesting that newly produced hair cell-like cells are biased to acquire outer hair cell specific characteristics in our experimental paradigm (Fig. 4 I, M).

### Newly formed hair cell-like cells are produced by supporting cells

**Figure 5:**
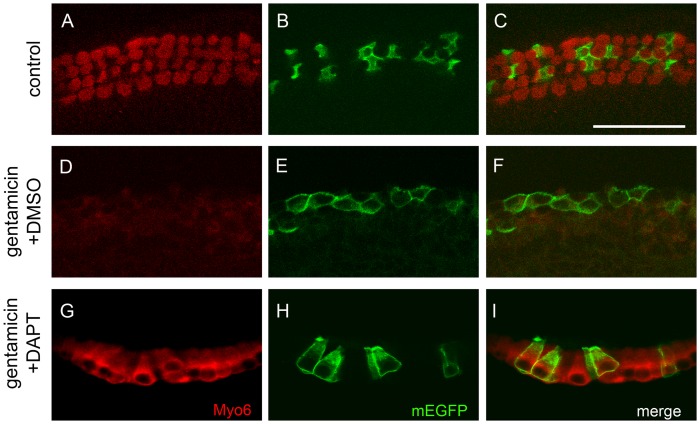
Newly generated hair cell-like cells originate from Prox1 positive supporting cells. P2 Prox1-CreER; mTomato/mEGFP transgenic control (A–C), gentamicin+ DMSO treated (D–F) and gentamicin +DAPT treated (G–I) cochlea explant cultures after 3 DIV. Note prior to plating tamoxifen injection at stage P0 and P1 permanently converted ∼ 10% of Prox1-CreER expressing supporting cells into membrane EGFP (mEGFP, green) positive cells. Myosin VI immuno-staining marks hair cells (Myo6, red). **A–C:** In control cultures, mEGFP positive supporting cells (B, C, green) surround myosin VI positive hair cells (A, C, red). **D–F:** Gentamicin treatment results in severe loss of myosin VI positive hair cells (D, F, red) and result in spread of mGFP positive supporting cells (E, F, green). **G–I:** In gentamicin and DAPT treated cultures mGFP positive cells (H, I, green) co-express hair cell specific marker myosin VI and acquire hair cell characteristic cell shape (G, I, red). Scale bar 50 μm.

Previous work demonstrated that early postnatal supporting cells retain the capacity to generate hair cells in dissociated culture [4]–[7]. To determine if the newly generated hair cell-like cells seen in our cultures originated from supporting cells, we cell-fate marked supporting cells utilizing the Prox1-CreER mouse strain [32] in combination with a mT/mG Cre reporter. The mT/mG Cre reporter expresses membrane-targeted tdTomato (mT) prior to, and membrane-targeted EGFP (mG or mEGFP) following, Cre-mediated recombination [33]. In the early postnatal cochlea, Prox1-CreER is expressed in inner pillar cells, outer pillar cells and Deiters cells [61]. Tamoxifen injection of Prox1-CreER; mT/mG transgenic pups at stage P1 and P2 resulted in the labeling of 10–20% supporting cells with mGFP (data not shown). mEGFP expression was strictly confined to supporting cells and no mEGFP positive hair cells were observed in our control cultures prior to- or at the end of the culture period (Fig. 5 B, C). Loss of hair cells due to gentamicin treatment caused mEGFP positive supporting cells to change cell shape and spread out, but no up-regulation of hair cell specific myosin VI was observed (Fig. 5 E, F). However, in gentamicin treated cultures, in which Notch signaling was blocked with GSI DAPT, a large fraction of mEGFP positive cells took on the cellular shape of hair cells and co-expressed the hair cell marker myosin VI (Fig. 5 H, I).

### Newly formed hair cell-like cells display hair cell-like rather than supporting-cell like electrophysiological properties

To determine whether newly generated hair cell-like cells exhibit hair cell-like or supporting-cell like electrophysiological properties, we next assessed some of their basic electrophysiological features using whole cell patch clamp recordings and compared them to control hair cells in untreated cultures as well as to supporting cells in untreated and treated cultures. These recordings were performed from the mid apical region of the cochlea to match the region analyzed by molecular biology and immuno-labeling methods. To estimate cell size, we measured cell capacitance Cm. At 6–7 DIV, hair cell-like cells in gentamicin +DAPT treated cultures had Cm of 7.8±0.4 pF (n = 11), similar to control inner hair cells (7.4±0.3 pF; n = 6) (One-way ANOVA followed by Bonferroni's *post hoc* test for multiple comparisons, *p* = 1) and slightly larger than control outer hair cells (5.6±0.6 pF; n = 8, p<0.012).

Hair cell-like cells in gentamicin +DAPT treated cultures did not show any difference regarding their input resistance Rm or their resting membrane potential Vm when compared to control hair cells (ANOVA, *p* = 0.3 and 0.7, respectively). The cell membrane resistance (Rm) of hair cell-like cells was 921±237 MΩ (n = 11), in comparison to 441±79 MΩ (n = 6) for inner hair cells and 1078±333 MΩ (n = 8) for outer hair cells. The resting membrane potential Vm of hair cell-like cells was −47.7±2.2 mV (n = 11), compared to −45.2±3.1 mV (n = 5) for inner hair cells and 43.2±6.5 mV (n = 6) for outer hair cells. Such Vm values show that the recorded hair cell-like cells as well as control hair cells were healthy, when recorded. Cm, Rm and Vm recorded here were consistent with hair cell properties recorded in earlier studies in acutely excised postnatal mouse cochleae [62], [63].

**Figure 6:**
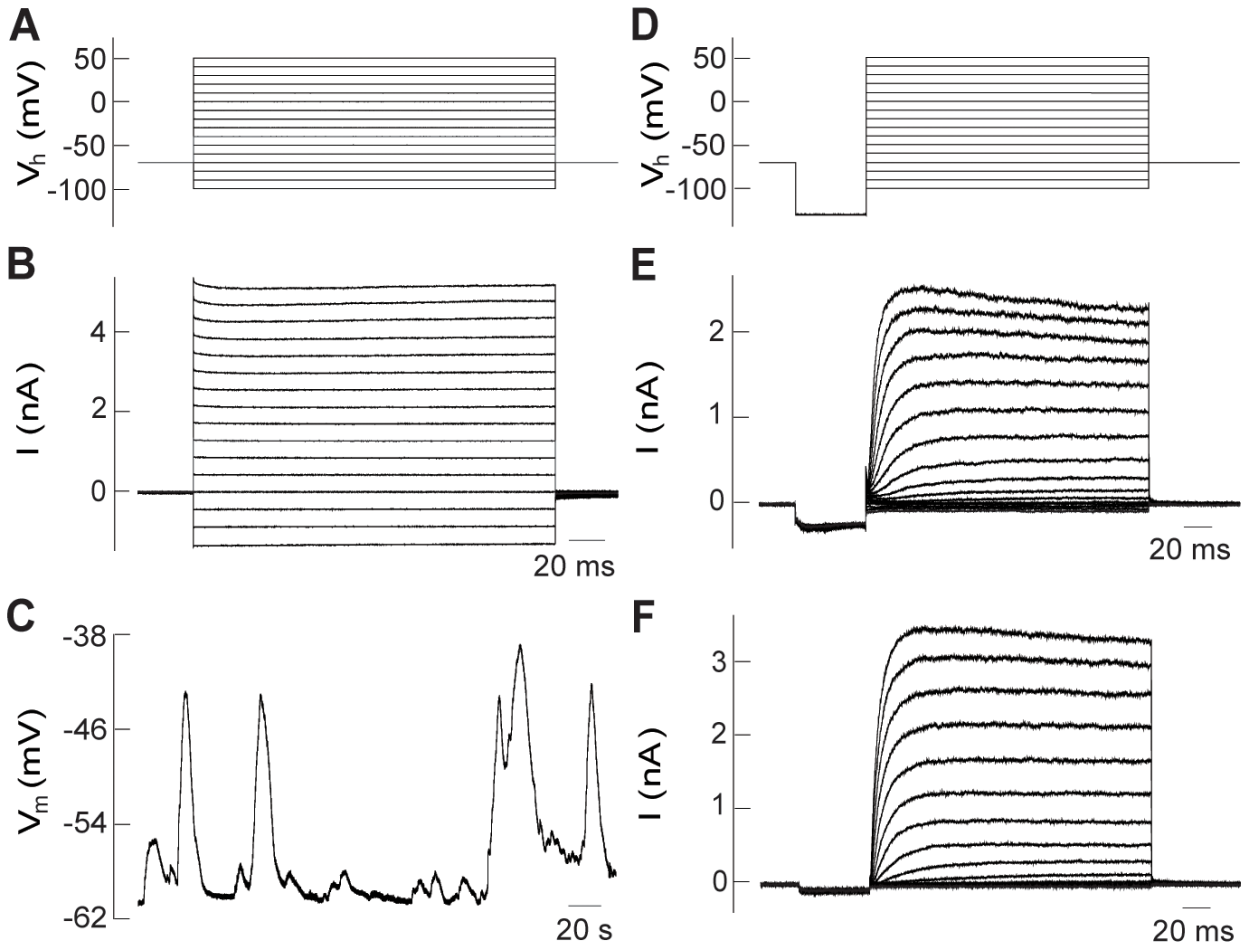
Newly formed hair cell-like cells have electrophysiological properties reminiscent of hair cells rather than supporting cells. **A**: Voltage step protocol as used in (***B***). From a holding potential of −70 mV, 200 ms voltage steps to values between −100 and 50 mV, in 10 mV increments were applied. **B**: Supporting cell recording, here from an untreated preparation after 6 DIV. Due to network coupling, supporting cells could not be voltage clamped and exhibited a typical ‘leaky’ response to a voltage step protocol. **C**: Supporting cells typically show resting membrane potentials that oscillate over a wide voltage range, here over ∼20 mV. **D**: Voltage step protocol as used in (***E***) and (***F***). From a holding potential of −70 mV, a 50 ms prestep to −130 mV was applied, followed by 200 ms voltage steps to values between −100 and 50 mV, in 10 mV increments. **E**: Recording from an Atoh1/nGFP positive, hair bundle bearing cell in a gentamicin +DAPT treated culture after 6 DIV shows delayed rectifier potassium currents qualitatively similar to control hair cells. **F**: Outer hair cell recording in an untreated preparation after 6 DIV shows delayed rectifier potassium currents.

Interestingly, hair cell-like cells as well as control hair cells showed clear differences when compared to supporting cells. Firstly, the Rm of supporting cells was 24.6±3.3 MΩ (n = 18), more than an order of magnitude smaller than Rm values of hair cell like-cells, or inner or outer hair cells (two tailed two sample t-tests for the individual hair cell groups versus supporting cells; *p*<0.016). This is mostly due to the coupling of supporting cells to a network of supporting cells via gap junctions [64]. Due to such network coupling, supporting cells could not be voltage clamped and exhibited a typical ‘leaky’ response to a voltage step protocol (Fig. 6 A, B). Secondly, supporting cell membrane potentials oscillated over a 20 to 30 mV range, a phenomenon that typically occurs in supporting cells before hearing onset [65] (Fig. 6 C). The average most-negative values for supporting cell membrane potentials during oscillation were −49.8±1.1 mV (n = 8) for gentamicin +DAPT treated and −64.2±1.4 mV (n = 10) for untreated cultures, assuring that recordings were performed from healthy cells. It has been shown earlier that the inner hair cell resting membrane potential is also capable of oscillating, but with a much smaller voltage range [65]. Due to the choice of our recording conditions here, no oscillations were found in control hair cells, which was also the case for hair cell-like cells.

To characterize voltage-gated conductances in hair cell-like cells, voltage step protocols were applied in voltage clamp. In contrast to supporting cells, postnatal rat and mouse hair cells have been shown to transiently express sodium channels [62], [66], [67]. To test for sodium channels, the voltage step protocol included a pre-step to a negative holding potential of −130 mV, to remove sodium channel inactivation, before 200 ms depolarizing voltage steps to values between −100 and 50 mV were applied (Fig. 6 D). Using this protocol, depolarizing voltage steps activated sodium currents in acutely isolated Atoh1/nGFP positive hair cells at P6 (data not shown), but failed to elicit sodium currents in cultured control hair cells (n = 14) or newly generated hair cell-like cells (n = 9) (Fig. 6 E, F). The lack of sodium currents suggests that the hair cell-like cells and control hair cells are either too immature to have developed sodium currents or that culture conditions interfere with the expression of sodium channels.

Although not an exclusive hair cell specific feature [68], slowly activating delayed rectifier potassium conductances typically occur in immature hair cells [62], [63], [69], [70]. Here, both hair cell-like cells (Fig. 6 E) and control hair cells (Fig. 6 F) showed similar current responses to the voltage step protocol, and activated delayed rectifier potassium currents, starting at depolarizing voltage steps to about −40 mV for the hair cell-like cells (n = 9) as well as for control hair cells (n = 14). Delayed rectifier currents were slowly activating and showed no or slight inactivation. For a voltage step from −130 mV to 0 mV, maximum current amplitudes varied widely for all groups tested without showing any significant differences (ANOVA, *p* = 0.4), with 855±185 pA (n = 9) for hair cell-like cells, compared to 1302±413 pA (n = 6) for inner hair cells and 853±196 pA (n = 8) for outer hair cells.

In summary, our data suggest that hair cell-like cells have basic electrophysiological properties similar to immature hair cells rather than supporting cells.

## Discussion

The long-term changes in supporting cell morphology and viability in the hair cell damaged cochlea are well documented [39], [71], [72]. However, much less is known about the acute effects of gentamicin exposure and hair cell loss on supporting cell specific gene expression, morphology, and viability. To characterize the molecular and cellular changes of supporting cells after acute hair cell loss we employed a well-characterized early postnatal cochlear culture system in combination with an optimized gentamicin based hair cell-ablation assay. The use of supporting cell specific Cre/loxP based labeling technique and GFP reporter line (Lfng/GFP) allowed us to monitor changes in supporting cell phenotype and behavior during and shortly after gentamicin insult. Our findings suggest that acute gentamicin based hair cell-depletion in the murine cochlea only modestly affects supporting cell viability, but induces a series of morphological changes in supporting cells. In avians, supporting cells engulf and phagocytose dying hair cells [73]. Similarly, in the acutely gentamicin damaged cochlear epithelia, hair cell membrane debris were found deep in the supporting cell layer, seemingly engulfed by supporting cells. After 3 DIV, hair cell debris were cleared from the epithelium and spreading supporting cells filled the voids left by hair cells. However, in contrast to avian auditory supporting cells, which re-enter the cell cycle in response to hair cell damage [2], [3], auditory supporting cells in the murine hair cell-depleted cultures failed to re-enter the cell cycle and remained postmitotic.

Recent studies demonstrated that Notch signaling continues to be active in the early postnatal supporting cells and genetic disruption or inhibition of Notch signaling using GSI results in robust production of ectopic hair cell-like cells in the early postnatal cochlea [23]–[25]. However, the role of Notch signaling in the hair cell-damaged cochlea is less clear. Using the experimental paradigm described here, we were able to examine how hair cell loss alters Notch effector and target expression in supporting cells. Our data suggest that in the early postnatal cochlea Notch signaling is only modestly weakened by the loss of hair cells, and identify Hes1, Hey1, Hey2, HeyL, and Sox2 as targets and likely Notch effectors of this hair cell-independent mechanism of Notch signaling. Moreover, we identify two distinct differences between Notch signaling in the intact and hair cell-ablated cochlea. First, Hes5 expression is almost completely lost after hair cell loss, suggesting that in the early postnatal cochlea, Hes5 expression is heavily dependent on hair cell-mediated Notch signaling. Second, our data suggest a different mechanism of regulation for Hes1 and Hey2 in the hair cell-depleted cochlea. In the intact auditory sensory epithelium, inhibition of Notch signaling does not significantly reduce Hes1 or Hey2 transcript levels [23], [24]. However, in the hair cell damaged cochlea, loss of Notch signaling results in significant reduction of Hey2 and Hes1 expression. The altered Notch responsiveness is likely due to the loss of hair cell-derived signals in the hair cell damaged cochlea. In the intact cochlea, Hey2 is co-regulated by Fgf and Notch signaling [24]. The loss of inner hair cell derived Fgf8 signaling in the hair cell damaged cochlea, likely disrupts Fgf signaling, rendering Hey2 Notch responsive.

The Notch ligand involved in this hair cell-independent mechanism of Notch signaling is unknown and experiments investigating the nature of the Notch ligand and its expressing cell type are in progress. One good candidate is the supporting cell-specific Notch ligand jagged1 (Jag1), which continues to be expressed by supporting cells even after extensive hair cell loss [27]. Alternatively, it is conceivable that DNER, or a yet uncharacterized Notch ligand expressed by nerve fibers of the innervating spiral ganglion could activate Notch receptors on contacting supporting cells [74].

It has been proposed that the newly formed hair cell-like cells seen after prolonged Notch inhibition are the result of supporting cells trans-differentiating into hair cells [24]. Employing Cre-loxP based labeling system, we are able to demonstrate that indeed, supporting cells are the source of the newly generated hair cell-like cells in our experimental paradigm. Moreover, we provide evidence that supporting cell specific features are rapidly down regulated and that these newly formed hair cell-like cells undergo a differentiation and maturation program, which resembles hair cell development *in vivo*. After 3 DIV, newly formed hair cell-like cells display gene expression profile characteristics of newly differentiated hair cells: they express “early” hair cell specific genes (e.g Atoh1, Pou4f3, Nhlh1, Fgf8, Otof, Myo6, Parv) but lack expression of “late” hair cell specific markers (Tmc1, Tmc2, Strc, Ocm, Slc26a5). After an additional 3 DIV (6 DIV), the *in vitro* generated hair cells up-regulate “late” hair cell specific genes and bear actin rich stereocilia-like hair cell bundles. After 6–7 DIV, hair cell-like cells showed electrophysiological properties similar to immature hair cells, rather than supporting cells. Supporting cells in untreated and gentamicin treated cultures had low membrane resistances (around 20 MΩ), due to their connections to neighboring supporting cells via gap junctions, and exhibited large membrane potential oscillations that occur throughout the network of supporting cells [65]. In contrast, hair cell-like cells as well as control hair cells had high membrane resistances of hundreds of MΩ and did not show membrane potential oscillations. These observations suggest that the newly differentiated hair cell-like cells have lost their functional coupling with their neighboring cells and possibly have down-regulated supporting cell specific programs and transformed into hair cell-like cells.

The molecular mechanisms that determine an inner versus an outer hair cell fate are largely unknown. Forced expression of Atoh1 in early postnatal Prox1 positive supporting cells converted these supporting cells into hair cell-like cells but these cells surprisingly failed to induce prestin expression or other outer hair cell specific characteristics [75]. In contrast, 90% of hair cell-like cells in Notch inhibited hair cell-ablated cultures expressed the outer hair cell specific protein prestin, indicating that regenerated hair cell-like cells differentiate predominantly into outer hair cell-like cells. The bias of newly generated hair cell-like cells towards an outer hair cell fate seen in the experimental paradigm described here should facilitate efforts to uncover the underlying molecular pathways involved in establishing outer and inner hair cell specific fates.

Recent studies in mice suggest that hair cell regeneration occurs in the adult vestibular utricle [76], [77]. The relative low rate of non-mitotic hair cell regeneration in the adult utricle can be significantly enhanced by treatment with GSI [78]. To date there is no evidence for spontaneous hair cell regeneration in the adult mammalian auditory sensory epithelium. However, there is growing evidence that mammalian auditory supporting cells have the intrinsic ability to function as hair cell progenitors. Once dissociated and cultured in a permissive environment, immature early postnatal auditory supporting cells generate hair cells through both mitotic and non-mitotic mechanisms [4], [5], [7]. Similarly, in the intact early postnatal cochlea, re-expression of the transcriptional activator Atoh1 [75], [79] and/ or Notch inhibition results in the generation of new hair cells [23]–[25]. Interestingly, these studies uncovered a difference in “hair cell generation competency” between the cochlear apex and cochlear base, with the highest number of new hair cells generated in the apex and the lowest number or no hair cells generated in the base. Similarly, our data demonstrate that supporting cells localized at the cochlear mid-apex readily trans-differentiate into hair cell like cells, whereas supporting cells in the cochlear base only infrequently do so. One possible explanation for the difference in apical and basal supporting cell competency might be the difference in supporting cell “age”. Cochlear differentiation occurs in a basal to apical gradient, with basal supporting cells differentiating first. It is possible that the apical to basal competence gradient mirrors an age dependent decline of supporting cell plasticity in the mammalian cochlea. Alternatively, it is possible that apical and basal supporting cells and/ or their environments are inherently different. Recent findings by Edge and colleagues demonstrate that in adult mice GSI-stimulated supporting cell to hair cell conversion only occurs in the cochlear apex but not in more basal regions of the cochlea [26]. Future studies are required to determine the molecular underpinnings for the “supporting cell competency gradient” in the mammalian cochlea.

In conclusion, our studies revealed that Notch signaling is still active in the hair cell damaged cochlea and we identify Hes1, Hey2, Hey1, HeyL, and Sox2 as Notch targets and likely Notch effectors of this hair cell-independent Notch signaling mechanism. Using a Cre/loxP based labeling system we demonstrate that loss of Notch signaling in the hair cell-damaged cochlea allows for supporting cells to trans-differentiate into hair cell-like cells. In addition, we demonstrate that these newly formed hair cell-like cells undergo a hair specific differentiation/maturation program, subtype specific specialization, and exhibit basic electrophysiological properties similar to early postnatal hair cells rather than supporting cells. We anticipate that the experimental paradigm described here will allow future discoveries of additional regulators and modulators of auditory supporting cell plasticity.

## Supporting Information

Figure S1 Hair cell phenotype in the acutely hair cell-damaged cochlea in the presence or absence of GSI DAPT. **A–B**: DAPT treatment does not protect hair cells from gentamicin toxicity. Atoh1/nGFP (green) expression reveals similar extend of hair cell loss in gentamicin + DAPT (B) and gentamicin +DMSO (A) treated cochlear explants after 1 DIV. Yellow arrow points to scattered Atoh1/nGFP positive hair cells, white arrow points to Atoh1/nGFP miss-expression in inner phalangeal cells. DMSO or DAPT was added after 14 hours of gentamicin treatment. Red lines subdivide auditory sensory epithelium into apex, mid apex, mid base and base. Scale bar 100 μm. **C–D:** Hair cell phenotype in gentamicin treated cochlear explants after 24 hours of DAPT (D) or DMSO (C) treatment. Shown are representative images of mid-apical region of the cochlea. Native Atoh1/nGFP expression (green) and myosin VI antibody staining (Myo6, red) marks hair cells. Only few scattered Atoh1/nGFP (green) Myo6 (red) double positive hair cells are present in the hair cell damaged cochlea after 24 hour DAPT (D) or DMSO (C) treatment. Yellow arrow points to scattered Atoh1/nGFP and Myo6 double positive hair cells, white arrow points to Atoh1/nGFP miss-expression in inner phalangeal cells. Scale bar 100 μm.
(TIF)
