# Supplementary figures and images for: Notch Signaling Limits Supporting Cell Plasticity in the Hair Cell-Damaged Early Postnatal Murine Cochlea
Source: PLoS One. 2013 Aug 30;8(8):e73276. doi: 10.1371/journal.pone.0073276 (PMC3758270; doi:10.1371/journal.pone.0073276)

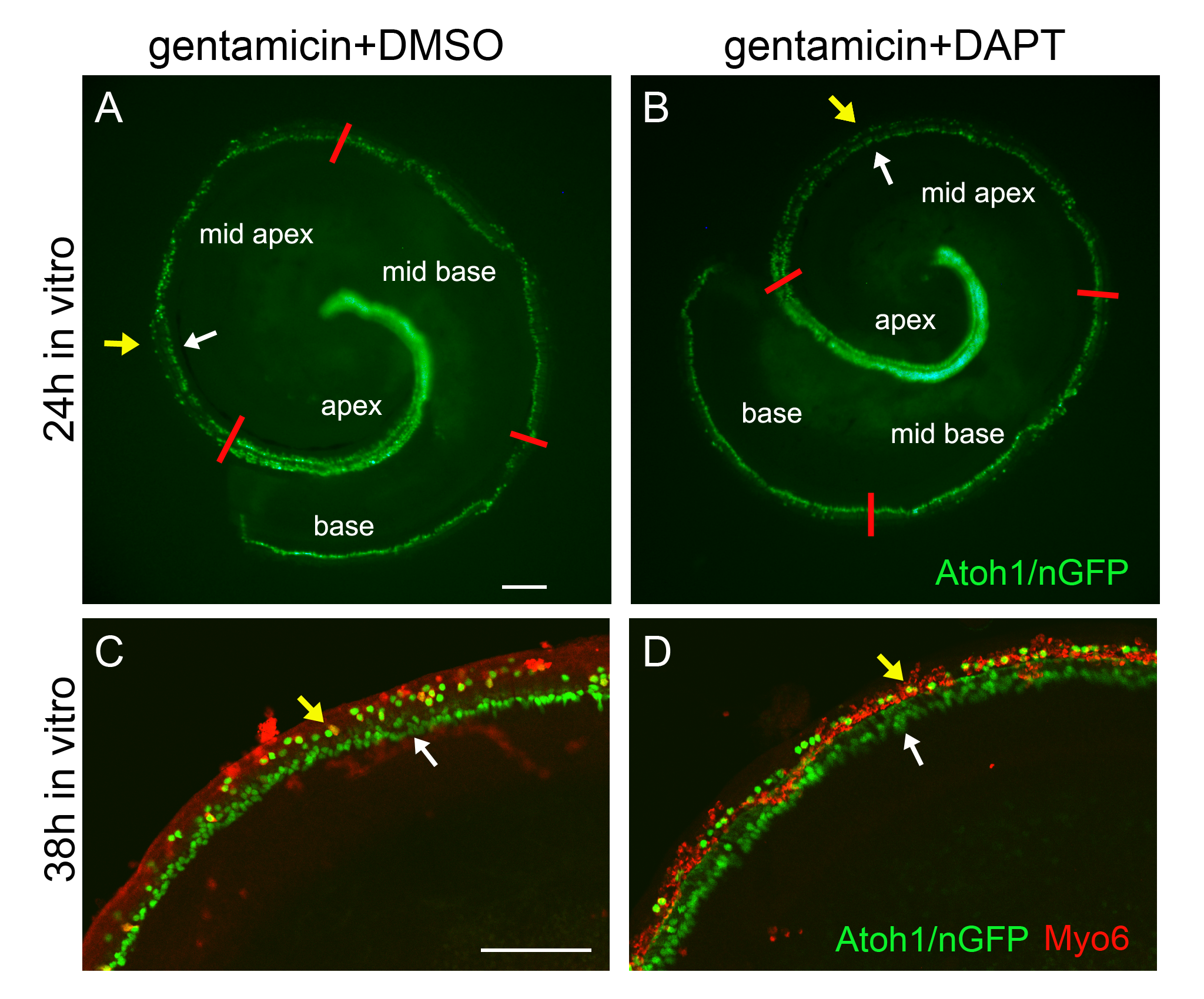

Supplement: Figure S1 — Hair cell phenotype in the acutely hair cell-damaged cochlea in the presence or absence of GSI DAPT. A–B: DAPT treatment does not protect hair cells from gentamicin toxicity. Atoh1/nGFP (green) expression reveals similar extend of hair cell loss in gentamicin + DAPT (B) and gentamicin +DMSO (A) treated cochlear explants after 1 DIV. Yellow arrow points to scattered Atoh1/nGFP positive hair cells, white arrow points to Atoh1/nGFP miss-expression in inner phalangeal cells. DMSO or DAPT was added after 14 hours of gentamicin treatment. Red lines subdivide auditory sensory epithelium into apex, mid apex, mid base and base. Scale bar 100 μm. C–D: Hair cell phenotype in gentamicin treated cochlear explants after 24 hours of DAPT (D) or DMSO (C) treatment. Shown are representative images of mid-apical region of the cochlea. Native Atoh1/nGFP expression (green) and myosin VI antibody staining (Myo6, red) marks hair cells. Only few scattered Atoh1/nGFP (green) Myo6 (red) double positive hair cells are present in the hair cell damaged cochlea after 24 hour DAPT (D) or DMSO (C) treatment. Yellow arrow points to scattered Atoh1/nGFP and Myo6 double positive hair cells, white arrow points to Atoh1/nGFP miss-expression in inner phalangeal cells. Scale bar 100 μm. (TIF) [file pone.0073276.s001.tif]
